# Excellent Response to Nivolumab and Ipilimumab in Metastatic Gastroesophageal Junction Squamous Carcinoma

**DOI:** 10.1155/2019/7405652

**Published:** 2019-07-11

**Authors:** Narayanan Sadagopan, Craig Devoe

**Affiliations:** ^1^Donald and Barbara Zucker School of Medicine at Hofstra/Northwell, Hempstead, NY, USA; ^2^Don Monti Division of Medical Oncology and Hematology, Northwell Health Cancer Institute, The Monter Cancer Center, Lake Success, NY, USA

## Abstract

Unresectable gastroesophageal junction (GEJ) cancers have a poor prognosis and limited treatment options. We report the case of a patient with a Siewert class III gastroesophageal junction squamous carcinoma with metastatic spread into the liver who had an exceptional response to a combination therapy of nivolumab and ipilimumab despite being programmed death-ligand 1 (PD-L1) negative, microsatellite stable (MSS), and having a low tumor mutational burden. He initially experienced disease progression on the chemotherapy regimens modified DCF and FOLFIRI resulting in limited functional status, esophageal stent placement, and feeding tube placement. After about 6 months on nivolumab and ipilimumab, he had near-complete disease resolution. He was able to return to his baseline functional status, as well as have the esophageal stent and feeding tube removed. Our case contributes to the value of exploring immunotherapy as an option for a variety of hard to treat cancers.

## 1. Introduction

Immunotherapy with checkpoint inhibitors continues to become a more common treatment option for a variety of cancers. Many studies have established the benefit of programmed cell death protein 1 (PD-1) and cytotoxic T-lymphocyte antigen-4 (CTLA-4) checkpoint inhibitors in the treatment of melanoma, renal cell carcinoma, and non-small cell lung cancer [1–3]. In most other cancers, the benefits of immunotherapy have been limited. Reports of excellent immunotherapy responses in squamous cell cancers of the head and neck and primary squamous cell cancers of the skin have been accumulating [4]. Squamous histology is not a common feature in primary gastric cancer representing 0.04% to 0.07% of all cases [5]. Despite FDA immunotherapy approvals for GEJ cancer that overexpress PD-L1 or are found to be microsatellite instable, the majority of GEJ cancers do not fit these criteria [6]. The CheckMate-032 study showed modest results for patients with metastatic GEJ cancer treated with nivolumab or a combination of nivolumab and ipilimumab. Response rates were numerically lower in immunologically cold tumors [7]. GEJ cancers are often diagnosed with regional lymph node involvement or metastatic disease making platinum and fluoropyrimidine-based systemic chemotherapy the initial treatment, even if the patient is going to undergo a surgical resection [8]. Trastuzumab is another agent often considered in metastatic GEJ cancers as 6.0% to 29.5% may overexpress human epidermal growth factor receptor 2 (HER2) [9]. Here, we present a case of a patient with a GEJ squamous carcinoma who experienced disease progression despite two different chemotherapy regimens, who then had an exceptional response to combination therapy of nivolumab and ipilimumab.

## 2. Case Presentation

**Figure 1:**
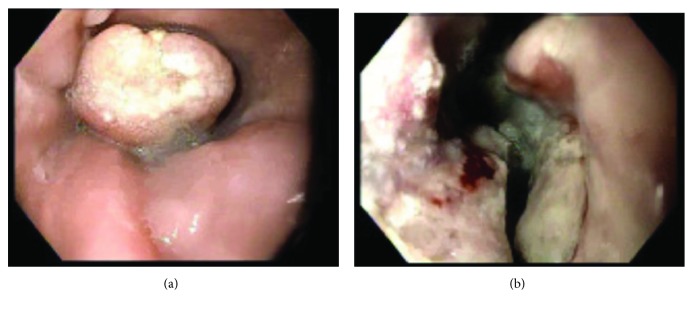
(a) Top of mass visualized on initial esophagogastroduodenoscopy. (b) Ulcerated portion of mass visualized on initial esophagogastroduodenoscopy.

The patient is a 50-year-old male who presented to the office in April 2017 for a 50-pound weight loss over the past year, fatigue, and difficulty swallowing solid foods. After initial imaging, an endoscopic ultrasound (EUS) showed a Siewert class III gastroesophageal junction mass extending from the top of the gastric folds to the body of the stomach (Figure 1), multiple bulky nearby lymph nodes with the largest lymph node being 5 cm, and a T3 N3 staging by the EUS criteria. Biopsy of the gastric mass revealed it was a high-grade carcinoma with glandular, neuroendocrine, and squamous differentiation. The tumor cells stained positive for the tumor markers pancytokeratin AE1/AE3, synaptophysin, p40, and CDX2 and did not overexpress HER2. An MRI in early May showed a 9 mm liver metastasis. He began treatment in May with modified DCF (docetaxel 40 mg/m^2^, cisplatin 40 mg/m^2^, and 5-fluorouracil 2000 mg/m^2^). Foundation One genomic alterations demonstrated the gene locations SMARCA4 P319fs^∗^7 and TP53 splice site 375+1G>A which were not actionable. Molecular markers for PD-L1 and microsatellite instability were negative, and the tumor mutation burden was low (4 mutations/megabase). In August, after he completed 6 cycles of modified DCF, chemotherapy was switched to FOLFIRI (leucovorin calcium, 5-fluorouracil, and irinotecan hydrochloride) due to mild disease progression on CT imaging and extreme fatigue from the modified DCF. Despite showing some initial response to the FOLFIRI on imaging, his FOLFIRI was held after 6 cycles in November due to severe nausea/vomiting and dehydration. He underwent multiple esophagogastroduodenoscopies (EGDs) later in November resulting in balloon dilatation and liquid nitrogen cryotherapy for a near-complete GEJ obstruction and eventually had an esophageal stent placed. He also had a percutaneous endoscopic gastrostomy (PEG) tube placed around this time since he could not tolerate eating by mouth. CT imaging in December showed worsening metastatic disease in the liver.

**Figure 2:**
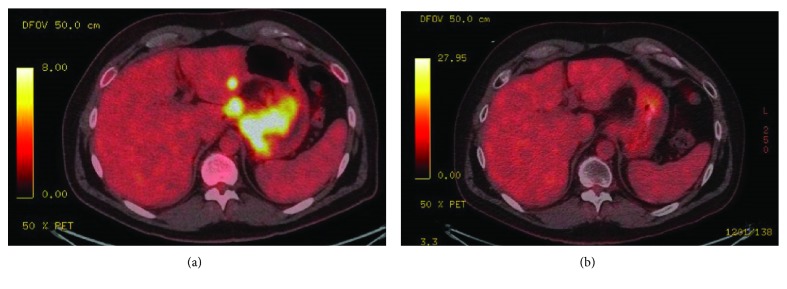
(a) Baseline axial fused PET/CT from May 2017 showing increased FDG uptake. (b) Axial fused PET/CT from March 2019 showing resolution of increased FDG uptake.

Based on the early data of CheckMate-032, the unique squamous histology of this patient's cancer, and the recent successes of PD-1 inhibition in other squamous carcinomas of the skin and head and neck, his treatment was switched to the off-label use of ipilimumab and nivolumab in December 2017. Due to his weakened condition and the known high concern for immune-related adverse events with the dual checkpoint blockade, the first 2 cycles were given with a reduced dose of ipilimumab at 1 mg/kg and nivolumab at 3 mg/kg. When he demonstrated good tolerance, this was followed by 4 cycles of ipilimumab at 3 mg/kg and nivolumab at 1 mg/kg. Also, at this time, he received 14 radiation treatments to the GEJ for obstruction. Over the next several months, he began to feel better, and his esophageal stent was removed at the end of March, as his scans showed improvement of the GEJ mass and metastatic hepatic lesions. His treatment was modified to monthly maintenance of nivolumab at a fixed dose of 480 mg starting in May 2018, and by June, he could eat without vomiting and was able to return to work full time. His PEG tube was removed in July. Later that month, a PET scan showed resolution of fluorodeoxyglucose (FDG) avidity in the GEJ, abdominal lymph nodes, and near total resolution in the hepatic metastases. He showed incredible improvement on ipilimumab and nivolumab with the main adverse effect being mild thyroiditis. His CT scan in November 2018 shows resolution of all visible disease, and a PET/CT scan in March 2019 was negative for the uptake of FDG isotope at all sites of disease (Figure 2). He will remain on monthly maintenance with nivolumab 480 mg until the end of the 2019.

## 3. Discussion

The patient's clinical course significantly changed from decreasing functional status and worsening tumor burden to near-complete resolution following treatment with nivolumab and ipilimumab. Since he also received radiation therapy during the initial part of the immunotherapy treatment, it is difficult to definitively say what role the radiation played in terms of the abscopal effect. The radiation likely contributed to the improvement of the patient's GEJ mass, but the continued improvement of nonradiated metastatic lesions in the months following the conclusion of the radiation therapy points to the substantial role of nivolumab and ipilimumab.

Interestingly, the patient's tumor was PD-L1 negative, had a low tumor mutational burden, and was MSS giving it features suggestive that cancer would be less likely to respond to immunotherapy. Immunotherapy biomarkers is an area where there is still a significant room for improvement to determine which patients will benefit from therapy. In this case, the rare squamous histology may have played a role given the recent success of immunotherapy in squamous cell cancers of the head and neck. It is possible that squamous cells have a feature that makes them more susceptible to immunotherapy. Alternatively, a PD-L1-positive status has some inherent variability. Some tumors may constitutively express PD-L1 while others only express it when T-cells infiltrate the tumor [10]. Thus, it is possible that the lack of T-cell infiltrate during sampling resulted in a false-negative PD-L1 status for our patient. While the exact mechanism of PD-L1 negative tumor response to nivolumab may be unknown, our patient who switched to only nivolumab after 6 cycles of combination immunotherapy continued to show improvement.

In conclusion, our case is an example of an exceptional response to immunotherapy in a patient who was running out of options after failing two chemotherapy regimens. He went from gastric tube feeds and an esophageal stent to keep his esophagus from collapsing to returning to his usual activities of daily living. His GEJ cancer responded spectacularly to immunotherapy despite having biomarkers suggestive of a poor response. A possible avenue for a future work would be to further investigate whether squamous histology is a feature in gastric cancer that indicates better response to immunotherapy. Our case contributes to the value of exploring immunotherapy as an option for a variety of hard to treat cancers.
